# miR-491-5p-induced apoptosis in ovarian carcinoma depends on the direct inhibition of both BCL-X_L_ and EGFR leading to BIM activation

**DOI:** 10.1038/cddis.2014.389

**Published:** 2014-10-09

**Authors:** C Denoyelle, B Lambert, M Meryet-Figuière, N Vigneron, E Brotin, C Lecerf, E Abeilard, F Giffard, M-H Louis, P Gauduchon, P Juin, L Poulain

**Affiliations:** 1 Normandie Univ, Caen, France; 2 UNICAEN BioTICLA Unit (Biology and innovative therapeutics of locally aggressive cancers) EA4656, Caen, France; 3 Comprehensive Cancer Center François Baclesse, UNICANCER, Caen, France; 4 CNRS, Délégation régionale Ile-de-France Est, 94532 Thiais cedex, France; 5 Team 8 Cell survival and tumor escape in breast cancer, UMR 892 INSERM/6299 CNRS, Université de Nantes, Institut de Recherche Thérapeutique de l'Université de Nantes, Nantes, France; 6 Institut de Cancérologie de l'Ouest (ICO), Centre de Lutte contre le Cancer René Gauducheau, Angers, France

## Abstract

We sought to identify miRNAs that can efficiently induce apoptosis in ovarian cancer cells by overcoming BCL-X_L_ and MCL1 anti-apoptotic activity, using combined computational and experimental approaches. We found that miR-491-5p efficiently induces apoptosis in IGROV1-R10 cells by directly inhibiting BCL-X_L_ expression and by inducing BIM accumulation in its dephosphorylated form. This latter effect is due to direct targeting of epidermal growth factor receptor (EGFR) by miR-491-5p and consequent inhibition of downstream AKT and MAPK signalling pathways. Induction of apoptosis by miR-491-5p in this cell line is mimicked by a combination of EGFR inhibition together with a BH3-mimetic molecule. In contrast, SKOV3 cells treated with miR-491-5p maintain AKT and MAPK activity, do not induce BIM and do not undergo cell death despite BCL-X_L_ and EGFR downregulation. In this cell line, sensitivity to miR-491-5p is restored by inhibition of both AKT and MAPK signalling pathways. Altogether, this work highlights the potential of miRNA functional studies to decipher cell signalling pathways or major regulatory hubs involved in cell survival to finally propose the rationale design of new strategies on the basis of pharmacological combinations.

Epithelial ovarian cancer is the leading cause of death from gynaecologic malignancies in women worldwide, causing over 140 000 deaths every year.^1^ Although progress has been made in its treatment by improved debulking surgery and the introduction of platinum–taxane regimens, the 5-year survival rate of advanced-stage epithelial ovarian cancer remains below 30%.^2^ This poor prognosis is mostly related to late diagnosis and chemoresistance. The identification of new molecular biomarkers and the development of individualized treatment regimens therefore appear as a major challenge for ovarian carcinoma therapeutic care.

Escape from apoptosis is an almost systematic hallmark of cancer cells that contributes to tumor progression and drug resistance.^3^ The BCL-2 family members constitute essential intracellular players in the apoptotic machinery.^4^ This family is composed of pro- and anti-apoptotic proteins sharing at least one of four BCL-2 homology domains (BH1 to 4). The balance between the antagonistic activities of these proteins determines mitochondrial outer membrane permeabilization and cell death decisions. BAX and BAK are essential effectors responsible for mitochondrial outer membrane permeabilization, whereas BCL-2, BCL-X_L_ and MCL1 preserve mitochondrial integrity. The third BCL-2 subfamily, BH3-only proteins (BIM, tBID, PUMA, BAD, NOXA, HRK) that sense cellular stress and are strictly regulated through both transcriptional and posttranslational mechanisms, promote apoptosis by either activating BAX and BAK (only for BIM, PUMA and tBID) and/or inactivating BCL-2, BCL-X_L_ or MCL1.

Altered expression and activity of BCL-2 family members are frequently found in cancer cells and contribute to an increased apoptotic threshold.^5^ Anti-apoptotic proteins of this family allow cancer cells to survive many stressful environments and cell death signals, such as those induced by oncogenic signals.^6^ Thus, BCL-2-like proteins represent a molecular vulnerability because inhibition of their survival activity may be sufficient to selectively eliminate cancer cells. In ovarian carcinoma, BCL-X_L_ and MCL1 are gateway proteins guarding together against apoptosis and their concomitant inhibition is sufficient to elicit apoptosis in chemoresistant ovarian cancer cell lines.^7, 8, 9^ On the basis of this assumption, the development of therapeutic strategies aiming at targeting concomitantly these two proteins could constitute an interesting alternative treatment of ovarian carcinoma. In this context, microRNAs (miRNAs) could represent an exciting field of interest to explore.

MiRNAs are small non-coding RNAs that negatively regulate gene expression either by inducing translational silencing or by causing mRNA degradation.^10^ MiRNAs have been shown to regulate many key cellular processes (i.e., proliferation, differentiation and apoptosis). With increasing research investigations, it is now becoming obvious that many miRNAs are misregulated in a variety of cancers,^11,12^ and influence the development and progression of cancer, including ovarian carcinoma.^13, 14, 15^ It has been shown that miRNAs can function as tumor promoters or tumor suppressors. Otherwise, one miRNA can regulate several hundreds of target mRNAs and, conversely, one mRNA can be targeted by multiple miRNAs. The interactions between miRNAs and their targets result consequently in the formation of complex regulatory networks, depending on the cellular context, related to cancer progression, cell survival, therapy resistance and metastasis. However, relatively few miRNA–target interactions have been experimentally validated, and the functions of a majority of miRNAs remain to be elucidated to provide novel therapeutic opportunities for cancer treatment.

In this study, using *in silico* prediction algorithms and functional studies, we attempted to uncover miRNA(s) that could induce apoptosis in ovarian cancer cells by targeting BCL-X_L_ and MCL1 and identify key signalling pathways involved.

## Results

### miR-491-5p induces apoptosis in IGROV1-R10 cell line and inhibits cell growth of SKOV3 cells

To identify potential miRNAs that could induce apoptosis in ovarian cancer cell lines, we performed an *in silico* search for putative miRNAs that could target BCL-X_L_ using miRNA target-prediction tools^16^ (Supplementary Figure S1). Among them, we focused on those that may also target MCL1. Each of the 11 selected miRNAs was transfected into two chemoresistant ovarian carcinoma cell lines (IGROV1-R10 and SKOV3) and cell proliferation was analysed (Supplementary Figure S2). We focused on miR-491-5p because it was found to be the most efficient cell growth inhibitor and was not expressed in both cell lines (personal data from miRNA profiling experiments).

**Figure 1:**
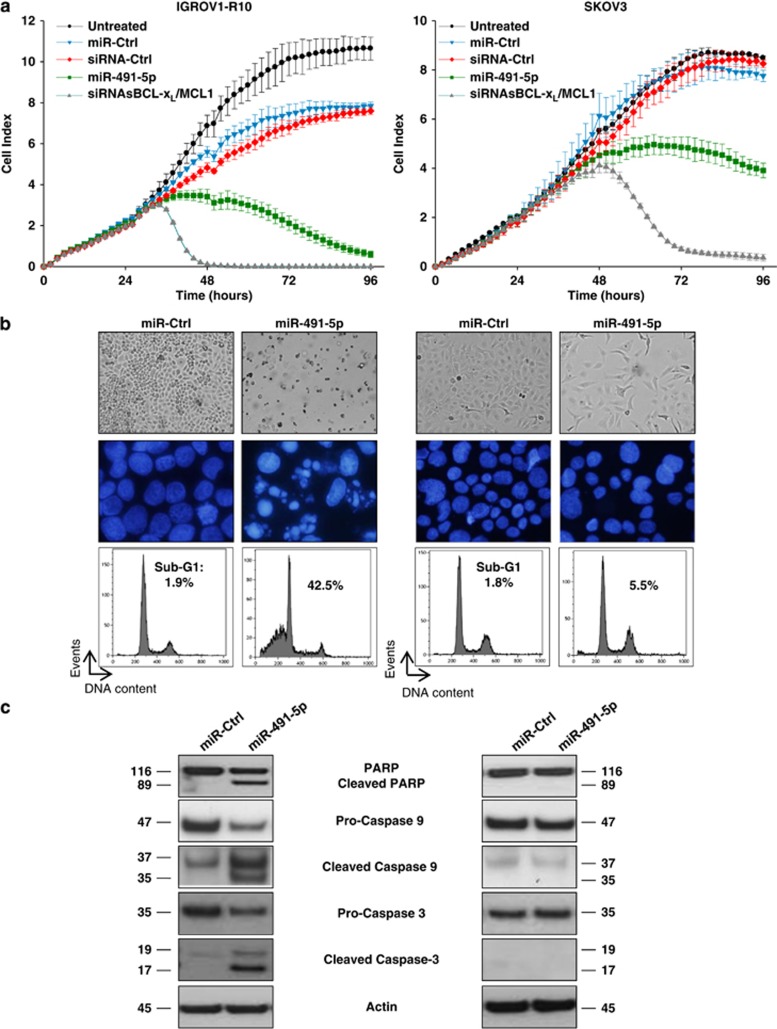
miR-491-5p induces apoptosis in IGROV1-R10 ovarian cancer cells and slow proliferation in SKOV3 ovarian carcinoma cell line. (**a**) Real-time growth curves monitoring was performed with the xCELLigence System. IGROV1-R10 (left panel) and SKOV3 (right panel) ovarian carcinoma cells were seeded into 96-well E-Plates VIEW, which contain electrodes integrated into the bottom surfaces of each well allowing the measurement of cell index (CI) based on impedance. CI correlates with the area of cells attached to the bottom of the plate. Cells were grown for 24 h before transfection with indicated RNAi (miRNA or siRNA). CI was recorded every 2 h for a time span of 96 h. The combination associating siRNAs targeting BCL-X_L_ (siRNA-BCL-X_L_) and MCL1 (siRNA-MCL1) was used as a positive control to visualize how cellular cytotoxicity could be visualized using the xCELLigence system. The experiment was performed three times in triplicates. Results are expressed as mean±S.D. on three independent experiments. (**b**) Cell morphology (upper column of each panel), nuclear morphology after DAPI staining (middle column of each panel) and DNA content histograms obtained by flow cytometry (lower column of each panel) were shown 72 h after transfection of IGROV1-R10 (left panel) and SKOV3 (right panel) ovarian carcinoma cells with miR-491-5p or miR-Ctrl (20 nM). (**c**) IGROV1-R10 (left panel) and SKOV3 (right panel) cells were transfected with miR-491-5p or miR-Ctrl. Cells were lysed after 72 h and protein extracts were assessed for levels of PARP (native and cleaved forms) and caspases-3 and -9 (pro and cleaved (active) forms) by western blotting. *β*-actin level is shown as loading control

The biological effect of miR-491-5p on both ovarian cancer cell lines was analysed in real-time with the xCELLigence system by following Cell Index (CI) over a period of 100 h. In IGROV1-R10 cells, miR-491-5p led to a drop of CI value at a similar extent than that observed with the combination of siRNAs targeting BCL-X_L_ and MCL1, but with delayed kinetics (Figure 1a). However, in SKOV3 cells, only a modest decrease in CI value was observed even at 96 h after treatment with miR-491-5p.

In good agreement with these results, we found that miR-491-5p induced apoptotic cell death in IGROV1-R10 cells as shown by massive pools of debris, the observation of numerous condensed or fragmented nuclei and the increase in the sub-G1 population (Figure 1b). Flow-cytometric analysis following Annexin V-FITC and propidium iodide staining also revealed that miR-491-5p induced apoptotic cell death (15.6% and 20.4% in early and late apoptosis, respectively), whereas miR-Ctrl had no effect (Supplementary Figure S3). In addition, the cleavage of caspases-3, -9 and PARP was clearly observed after transfection with miR-491-5p (Figure 1c). In contrast, these events were not observed in SKOV3 cells. Altogether, these results indicate that miR-491-5p induced a cytotoxic effect on IGROV1-R10 cells, whereas it induced a cytostatic effect without associated apoptosis in SKOV3 cells.

### miR-491-5p directly inhibits BCL-X_L_ expression (but does not target MCL1)

**Figure 2:**
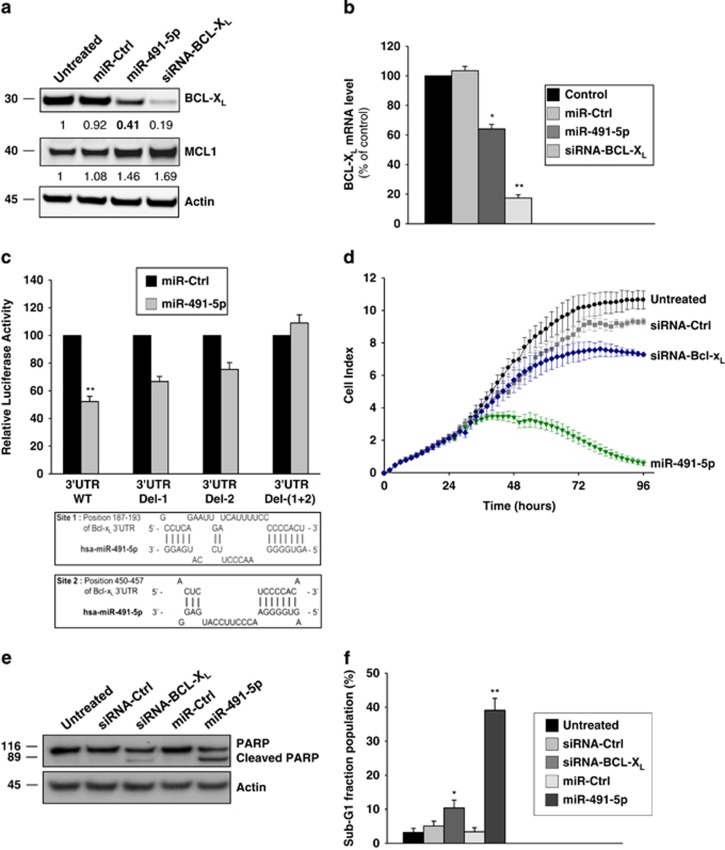
miR-491-5p directly targets BCL-X_L_ and may regulate additional targets involved in miRNA-mediated cell death in IGROV1-R10 cells. (**a**) IGROV1-R10 ovarian chemoresistant cell line was transfected with indicated RNAi (20 nM). BCL-X_L_ and MCL1 protein expression levels were determined by western blot 72 h after transfection. The relative densitometry values (calculated relative to *β*-actin) were determined using ImageJ software and are shown on the bottom of each western blot. (**b**) Relative quantification in real-time PCR of BCL-X_L_ mRNA levels in IGROV1-R10 cells after transfection with 20 nM indicated miRNA mimics or with siRNA targeting BCL-X_L_ (siRNA-BCL-X_L_). Data are normalized to GAPDH mRNA levels as an endogenous control. Results are expressed as mean±S.D. of three independent experiments. (**c**) Schematic representation of 3′-UTR *BCL-X*_*L*_ mRNA with two predicted binding sites of hsa-miR-491-5p. Luciferase reporter constructs containing human *BCL-X*_*L*_ 3′-UTR wild-type (WT) or mutant deletion of putative binding site 1, site 2 or both sites for miR-491-5p were transfected into SKOV3 cells concomitantly with miR-491-5p or the control miRNA (miR-Ctrl). Luciferase assays were carried out 24 h after transfection. Firefly luciferase activity was normalized to renilla luciferase. Data are presented as mean±S.D. of three independent experiments. (**d**) Real-time curve monitoring growth of IGROV1-R10 with the xCELLigence System. Cells were grown for 24 h before transfection with indicated RNAi (miRNA or siRNA). CI was recorded every 2 h for a time span of 96 h. The experiment was performed three times in triplicates; shown is mean±S.D. on representative experiment. (**e**) IGROV1-R10 cells were transfected with indicated RNAi. Seventy-two hours after transfection, cells were subjected to flow cytometry to determine the percentage of sub-G1 population. For each condition, the percentage of sub-G1 population, representative of three independents experiments, is indicated. (**f**) Protein expression levels of PARP (native and cleaved forms) were determined by western blot 72 h after transfection of IGROV1-R10 cells with indicated RNAi

We next wanted to determine whether miR-491-5p-induced apoptosis in IGROV1-R10 cells could be related to the inhibition of both BCL-X_L_ and MCL1. We observed that BCL-X_L_ mRNA and protein levels were decreased in IGROV1-R10 cells transfected with miR-491-5p, whereas no clear changes were observed for miR-Ctrl-transfected cells (Figures 2a and b). Although computational analysis predicted that MCL1 could be a potential target of miR-491-5p, we found that miR-491-5p did not decrease but rather increase the level of MCL1 protein in IGROV1-R10 cell line (Figure 2a). We also analysed whether the reduced expression of BCL-X_L_ was due to direct targeting by miR-491-5p. Computational analysis allowed to identify two high-scoring putative target sites for miR-491-5p in the 3′-untranslated region (UTR) of human *BCL-X*_*L*_ (Figure 2c). Co-transfection of wild-type *BCL-X*_*L*_ 3′-UTR reporter construct with miR-491-5p mimic substantially decreased the luciferase activity in transfected cells. Additionally, although each individual deletion resulted only in a weak repression of luciferase activity, deletion of both binding sites abolished miR-491-5p-induced decrease of luciferase activity. These results demonstrate that miR-491-5p represses BCL-X_L_ protein expression through direct binding to these two binding sites in 3′-UTR of the human *BCL-X*_*L*_. Finally, although miR-491-5p targets only one of the two proteins initially demonstrated as essential for the protection against apoptosis (BCL-X_L_ and MCL1), its apoptotic effect was shown to be more important than the simple inhibition of BCL-X_L_ by an siRNA (Figures 2d–f). These observations suggest that additional targets might be involved in miR-491-5p-mediated cell death.

### Induction of the BH3-only protein BIM by miR-491-5p is critical for the induction of apoptosis in IGROV1-R10 cells

**Figure 3:**
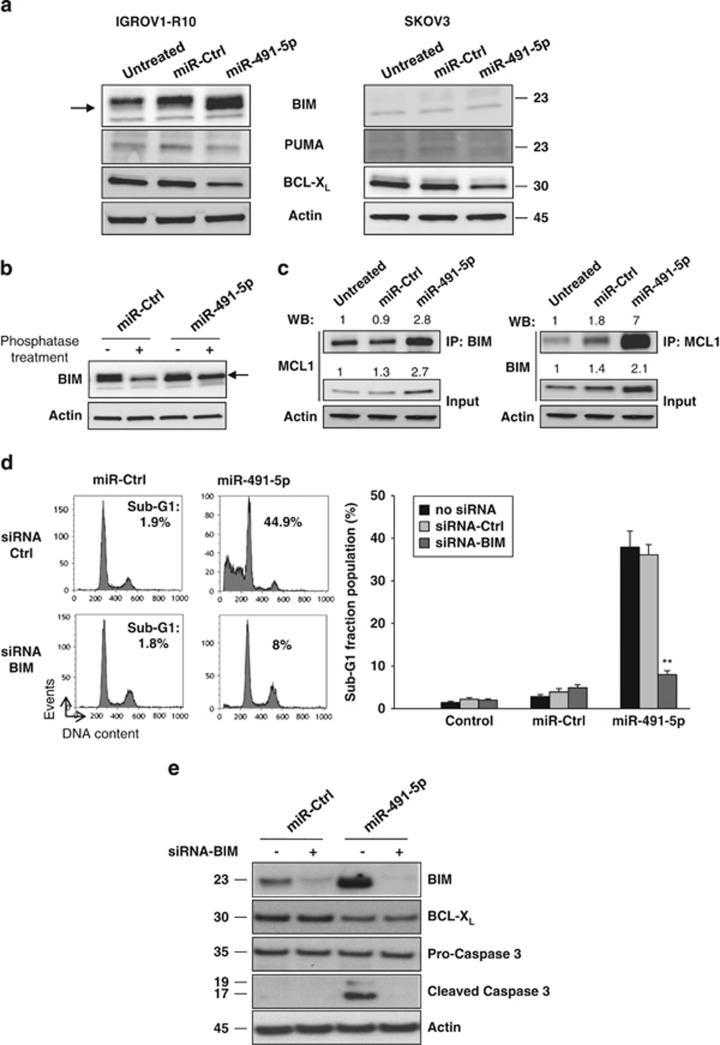
BIM is induced by miR-491-5p and is a critical determinant in miRNA-mediated apoptosis in IGROV1-R10 cells. All data presented were obtained 72 h after transfection of IGROV1-R10 or SKOV3 cells with miR-491-5p or miRNA-Ctrl (miR-Ctrl). (**a**) Protein expression levels of the BH3-only BIM and PUMA and the anti-apoptotic BCL-X_L_ were assessed by western blotting. Changes in migration on SDS-PAGE of BIM were indicated with an arrowhead. (**b**). Lysates from miR-Ctrl or miR-491-5p transfected IGROV1-R10 cells were treated or not with phosphatase alkaline and analysed by western blotting for changes in migration of BIM in SDS-PAGE. Changes in phosphorylation on SDS-PAGE of BIM were indicated with an arrowhead. (**c**) IGROV1-R10 cells were harvested, lysed and subjected to immunoprecipitation (IP) with BIM or MCL1 antibodies. The resulting immune complexes as well as total lysates were analysed by western blotting with BIM or MCL1 antibodies. The relative densitometry values (calculated relative to control conditions after normalization with *β*-actin) were determined using ImageJ software and are shown on the bottom of each western blot. (**d** and **e**) IGROV1-R10 cells were transfected concomitantly with siRNA against BIM (siRNA-BIM) or siRNA control and indicated miRNAs. (**d**) Seventy-two hours after transfection, cells were subjected to flow cytometry to determine the percentage of sub-G1 population (data are presented as mean±S.D. of triplicate experiments). A representative DNA content histogram obtained by flow cytometry is also shown. (**e**) Cells were lysed and subjected to western blot analysis to determine BIM, BCL-X_L_, caspases-3 (pro and cleaved forms) protein expression levels. *β*-actin level is shown as loading control

We next explored whether miR-491-5p-induced apoptosis in IGROV1-R10 cells involved pan inhibitor BH3-only proteins BIM and PUMA.^4^ We first observed that miR-491-5p did not change PUMA expression in IGROV1-R10 cells (Figure 3a and Supplementary Figure S4). In contrast, the level of BIM was strongly increased in response to miR-491-5p in IGROV1-R10 cells, whereas it remained unchanged after transfection with an miRNA control or siRNA targeting BCL-X_L_ (Supplementary Figure S7). Furthermore, we observed that miR-491-5p downregulates BCL-X_L_ but does not increase BIM in the apoptotic resistant SKOV3 cells, suggesting that the differential effect of the miRNA observed between the IGROV1-R10 and SKOV3 cell lines may be related to the induction or the default of induction of BIM, respectively.

We showed that BIM underwent a mobility shift after transfection of miR-491-5p in IGROV1-R10 cells (Figure 3a). Treatment of protein extracts from control IGROV1-R10 cells with calf-Intestinal alkaline phosphastase resulted in mobility shifts of BIM that were similar to those observed in BIM from extracts of IGROV1-R10 cells transfected with miR-491-5p (Figure 3b). This observation indicated that BIM is dephosphorylated in response to miR-491-5p. This finding is notable because dephosphorylation of BIM results in an increase in its pro-apoptotic function.^17^ In addition, immunoprecipitation studies revealed that the amount of BIM bound to MCL1 increases drastically after transfection of miR-491-5p in IGROV1-R10 cells suggesting an increase of sequestration of MCL1 by BIM (Figure 3c). Finally, we observed that BIM silencing confers a marked resistance of IGROV1-R10 cells to miR-491-5p-induced apoptosis as shown by both a minor sub-G1 peak on DNA histograms and the absence of caspase-3 cleavage (Figures 3d and e). Altogether, these observations underline the critical involvement of BIM in miR-491-5p-induced cell death.

### BIM stabilization induced by miR-491-5p is related to direct targeting of the epidermal growth factor receptor (EGFR) and subsequent AKT and MAPK pathways inhibition in IGROV1-R10 cells

**Figure 4:**
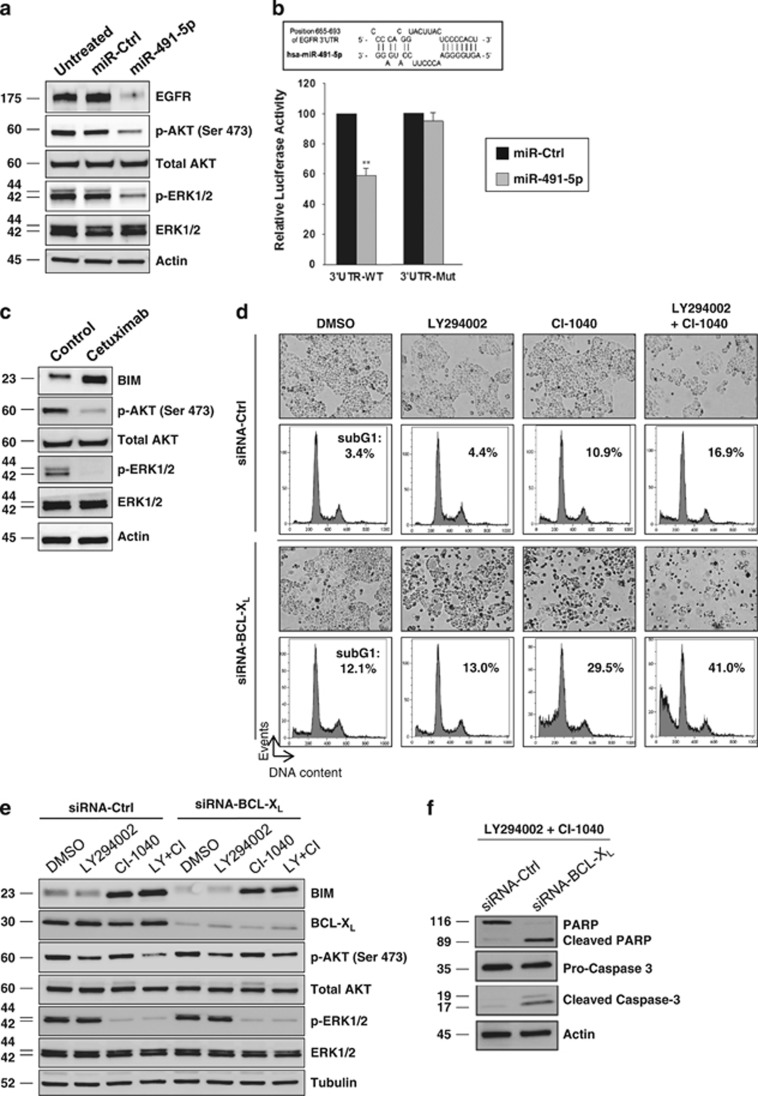
miR-491-5p by targeting EGFR downregulates AKT and MAPK pathways leading to BIM induction and dephosphorylation in IGROV1-R10 cells. (**a**) IGROV1-R10 cells were transfected with miR-491-5p or miR-Ctrl for 72 h. Total cell extracts were assessed for levels of both phosphorylated AKT (p-AKT) and ERK (p-ERK), both total AKT and ERK and EGFR by western blotting. *β*-actin level is shown as loading control. (**b**) Schematic representation of 3′-UTR *EGFR* mRNA with its predicted binding sites of hsa-miR-491-5p. Luciferase reporter constructs containing human *EGFR* 3′-UTR wild-type (WT) or mutant deletion of putative binding site for miR-491-5p were transfected into SKOV3 cells concomitantly with miR-491-5p or the control miRNA (miR-Ctrl). Luciferase assays were carried out 24 h after transfection. Firefly luciferase activity was normalized to renilla luciferase. Data are presented as mean±S.D. of three independent experiments. (**c**) IGROV1-R10 cells were treated with cetuximab (100 *μ*g/ml) for 72 h and protein extracts were analysed by western blot with indicated antibodies. (**d** and **e**) IGROV1-R10 cells were transfected with siRNA against BCL-X_L_ (siRNA-BCL-x_L_) or siRNA control (siRNA-Ctrl) followed by treatment with DMSO or with 7.5 *μ*M LY294002 (PI3K inhibitor) or 2 *μ*M CI-1040 (MEK inhibitor) or both pharmacological inhibitors (LY294002+CI-1040) for 48 h. (**d**) Representative cell morphology and DNA content histograms obtained by flow cytometry are shown. The percentage of cells in sub-G1 phase was indicated for each condition. Similar results were obtained in three independent experiments. (**e**) Protein lysates were immobloted with the indicated total and phospho-specific antibodies. *β*-actin level is shown as loading control. (**f**) IGROV1-R10 cells were transfected with siRNA against BCL-X_L_ (siRNA-BCL-X_L_) or siRNA control followed by a treatment with LY294002+CI-1040 for 48 h. Whole-cell lysates were analysed by western blotting for PARP (native and cleaved forms) and caspase-3 (pro and cleaved (active) forms)

Accumulating evidences have revealed that EGFR inhibitors can lead to suppression of AKT and MAPK pathways, followed by the increase in expression and dephosphorylation of pro-apoptotic BIM in different cellular contexts.^18^ This led us to focus our attention on the EGFR signalling pathway. Indeed, using computational analysis, we noticed that *EGFR* was among the top-rank predicted target of miR-491-5p. We found that miR-491-5p induces an important decrease of EGFR protein level in IGROV1-R10 cells and diminishes activation of both AKT and MAPK pathways (Figure 4a). Importantly, we demonstrated that miR-491-5p binds directly to the *EGFR* 3′-UTR to mediate the repressive effect on protein expression. Indeed, co-transfection of *EGFR* wild-type 3′-UTR reporter construct with the miR-491-5p mimic reduced the relative luciferase activity. Moreover, deletion of the miR-491-5p binding site in the 3′-UTR of *EGFR* completely abrogated the inhibitory effect induced by miR-491-5p (Figure 4b).

Next, we observed that cetuximab (a monoclonal antibody inhibitor of EGFR) decreased AKT and ERK phosphorylation and led to BIM induction and dephosphorylation (Figure 4c). In addition, treatment of IGROV1-R10 cells with two specific inhibitors to block both PI3K (LY294002) and MEK (CI-1040) led to BIM stabilization (Figure 4e). This strongly suggests that EGFR inhibition by miR-491-5p and subsequent reduced activation of both AKT and MAPK pathways led to BIM stabilization in IGROV1-R10 cells. Finally, we showed that both pharmacological inhibitors in combination with a siRNA targeting BCL-X_L_ led to apoptosis at a similar extent than miR-491-5p (Figures 4d–f). Altogether, these results demonstrate that the efficiency of miR-491-5p to mediate cell death in IGROV1-R10 cells is critically related to its ability to inhibit, on one hand, both AKT and MAPK signalling pathways downstream of EGFR (for efficient BIM accumulation) and, on the other hand, BCL-X_L_ expression.

### Dual inactivation of PI3K/AKT/mTOR and MAPK signalling pathways are required to induce BIM expression and dephosphorylation in SKOV3 cells

**Figure 5:**
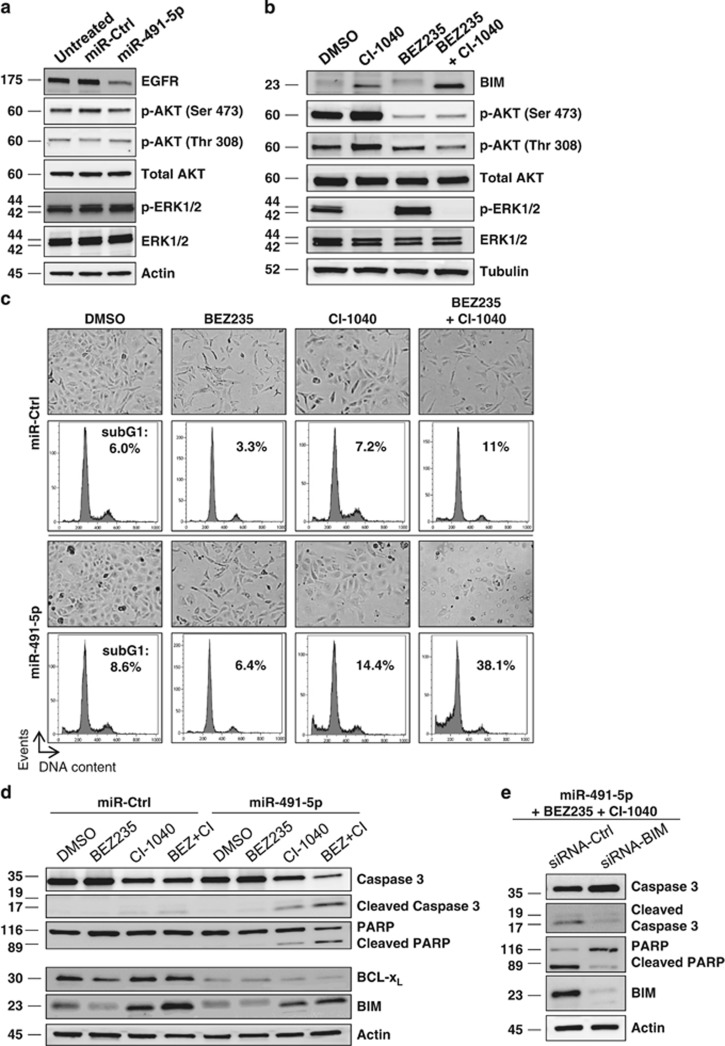
Combining BEZ235 with CI-1040-induced BIM expression and sensitizes SKOV3 cell line to miR-491-5p. (**a**) SKOV3 cells were transfected with miR-491-5p or miR-Ctrl for 72 h. Whole-cell extracts were analysed by western blotting with the indicated total and phospho-specific antibodies. *β*-actin level is shown as loading control. (**b**) SKOV3 cells were treated with DMSO or 5 *μ*M CI-1040 (MEK inhibitor) or 1 *μ*M BEZ235 (a dual PI3K and mTOR inhibitor) or with both pharmacological inhibitors (BEZ235 and CI-1040) for 72 h. Whole-cell lysates were then subjected to western blot analysis with the indicated total and phospho-specific antibodies. *β*-actin level is shown as loading control. (**c** and **d**) SKOV3 cells were transfected with miR-491-5p or miR-Ctrl followed by treatment with DMSO or with BEZ235 or CI-1040 or both pharmacological inhibitors (BEZ235+CI-1040) for 72 h. (**c**) Representative cell morphology and DNA content histograms obtained by flow cytometry are shown. The percentage of cells in sub-G1 phase was indicated for each condition. Similar results were obtained in three independent experiments. (**d**) Protein lysates were immunobloted with the indicated total and phospho-specific antibodies. *β*-actin level is shown as loading control. (**e**) SKOV3 cells were co-transfected with miR-491-5p and siRNA against BIM (siRNA-BIM) or siRNA control followed by a treatment with BEZ235+CI-1040 for 72 h. Whole-cell lysates were analysed by western blotting for PARP (native and cleaved forms) and caspase-3 (pro and cleaved (active) forms)

**Figure 6:**
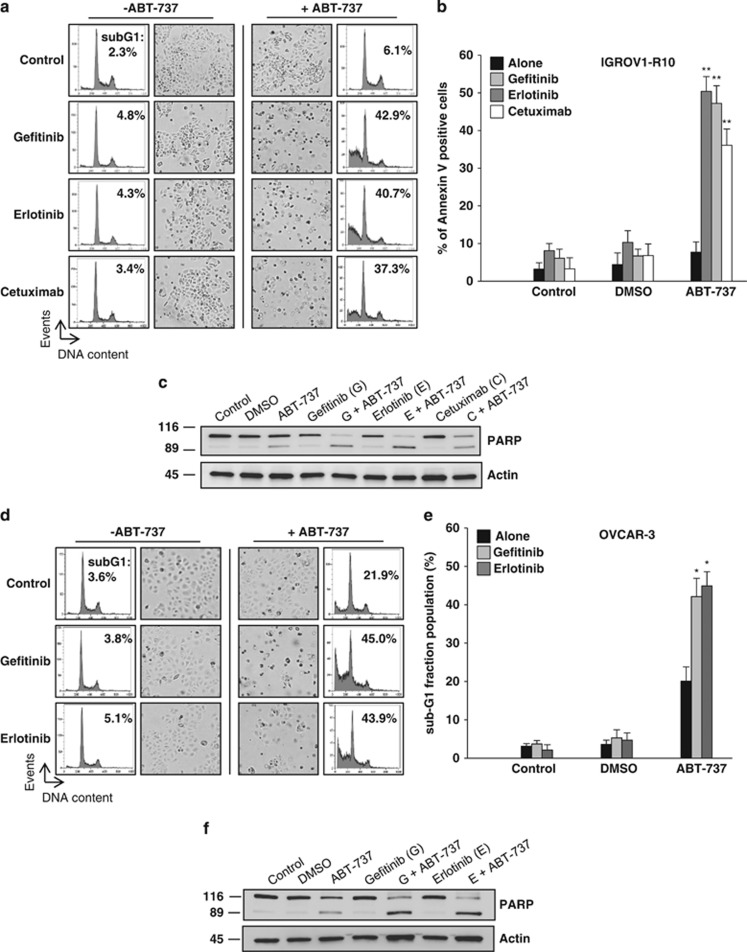
The combination of an EGFR inhibitor and a BH3-mimetic molecule (ABT-737) induces cell death in IGROV1-R10 and OVCAR-3 ovarian cancer cell lines. For all experiments, IGROV1-R10 and OVCAR-3 cells were treated for 48 h with 100 *μ*g/ml of cetuximab, 1 *μ*M erlotinib, 1 *μ*M gefitinib in the presence or the absence of 5 *μ*M ABT-737. (**a** and **d**) Cell morphology and DNA content histograms obtained by flow cytometry were shown following 48 h of treatment with indicated drugs in IGROV1-R10 (**a**) and OVCAR-3 (**d**) cells. (**b**) The fraction of cells positive for Annexin V was determined by flow cytometry 48 h after treatment with indicated drugs. Data are expressed as mean±S.D. of three independent experiments. (**c**) Whole-cell lysates from IGROV1-R10 cells were subjected to western blot analysis of PARP (native and cleaved forms). *β*-actin level is shown as loading control. (**e**) The fraction of OVCAR-3 cells with sub-G1 DNA content was determined by flow cytometry after PI staining. Data are expressed as mean±S.D. of three independent experiments. (**f**) Protein lysates from OVCAR-3 cells were immunobloted with the PARP (native and cleaved forms) antibody. *β*-actin level is shown as loading control

We showed that miR-491-5p induced a decrease of EGFR and BCL-X_L_ protein levels in SKOV3 cells as previously observed in IGROV1-R10 cells (Figure 5a). However, miR-491-5p (Figure 5a) and cetuximab (Figure 6b) failed to inhibit both ERK and AKT activity in SKOV3 cells, suggesting that some alterations downstream of EGFR may be responsible for the sustained activation of both cascades and subsequent BIM degradation. We observed that the level of ERK activation is strongly reinforced in the SKOV3 cell line as compared with the IGROV1-R10 cell line (Supplementary Figure S5). Furthermore, previous studies have shown that SKOV3 cells harbour an activating mutation (H1047R) in the *PIK3CA* gene, which results in the continuous phosphorylation of AKT.^19^ As it was recently shown that a dual PI3K and mTOR inhibitor is required to downregulate efficiently AKT cell signalling in SKOV3 cells,^20^ we chose to work with BEZ235 rather than LY294002. We found that the BEZ235/CI-1040 combination treatment led to a significant increase in expression and dephosphorylation of BIM suggesting that the inhibition of both PI3K/AKT/mTOR and MAPK pathways is required to stabilize BIM in SKOV3 cells as previously observed in IGROV1-R10 cells (Figure 5b). Of note, the expression of BIM was unchanged (with BEZ235) or slightly increased (with CI-1040) after treatment by each pharmacological inhibitor as single agent.

### Combining BEZ235/CI-1040 treatment induces BIM expression and sensitizes the SKOV3 cell line to miR-491-5p

We investigated whether restoration of BIM expression in the SKOV3 cell line could confer sensitivity to miR-491-5p. Indeed, although BEZ235/CI-1040 treatment associated with a control miRNA did not display any cytotoxicity, the same treatment in combination with miR-491-5p (or siRNA-BCL-X_L_^21^) induced a marked apoptosis in the SKOV3 cell line (Figures 5c and d and Supplementary Figure S6a). We did not see substantial responses to the combination of miR-491-5p with either CI-1040 or BEZ235 alone. Finally, we observed that BIM silencing protected SKOV3 cells from BEZ235/CI-1040/miR-491-5p-induced apoptosis (Figure 5e and Supplementary Figure S6b), suggesting that the BIM stabilization is critical to induce cell death in this cell line.

### EGFR inhibitor and the BH3-mimetic molecule ABT-737 synergize in the killing of ovarian cancer cell lines

We wondered whether the association of an anti-EGFR monoclonal antibody (cetuximab) or EGFR TKIs (erlotinib and gefitinib) with a BH3-mimetic molecule (ABT-737), which binds and neutralizes BCL-X_L,_ could have the same effect as miR-491-5p. Indeed, the combination of these different EGFR inhibitors with ABT-737 (or siRNA-BCL-X_L_) induced apoptosis in IGROV1-R10 cells, whereas each drug alone had no obvious effect (Figures 6a–c and Supplementary Figure S7). Moreover, we observed that this combination could be also effective in the ovarian cancer cell line OVCAR-3. Indeed, whereas ABT-737 induced a mild apoptosis, the combination of EGFR TKI (erlotinib or gefitinib) with a BH3-mimetic molecule led to a massive apoptosis in OVCAR-3 cells (Figures 6d–f). Altogether, these observations highlight the interest in pursuing a combination therapy of an EGFR inhibitor with a BH3-mimetic molecule in ovarian cancer. Finally, as expected with our previous results obtained with miR-491-5p, the combination of cetuximab (or erlotinib or gefitinib) and ABT-737 (or siRNA-BCL-X_L_) did not induce cell death in SKOV3 cells (Supplementary Figure S8).

## Discussion

The poor outcome of patients diagnosed with ovarian carcinoma and treated with conventional chemotherapy emphasizes the urgent need to develop new therapeutical strategies. As the involvement of miRNAs in ovarian oncogenesis and progression has started to emerge recently, studies are needed to identify their molecular targets and the processes they affect, and to define new key pathways or networks that could finally lead to the development of new targeted therapeutic strategies.

We evidenced that miR-491-5p induces apoptosis and inhibits proliferation of ovarian carcinoma cells. Interestingly, other studies have also reported the similar effects in colorectal,^22^ pancreatic^23^ and breast cancer cells.^24^ In addition, an inhibitory effect of miR-491-5p on invasion of glioma,^25^ breast^26^ and oral squamous^27^ cancer cells was also described, suggesting that miR-491-5p functions as a '*bona fide*' tumor suppressor. In ovarian cancer, miR-491-5p has been reported to be downregulated both in the late-stage cancer in comparison with the early-stage^28^ and in non-responder *versus* responder^29^ cancer patients. Our study highlights that miR-491-5p reintroduction could be of interest for the treatment of ovarian cancer patients. However, although a liposome-based miR-34 mimic recently entered in phase I clinical trial in patients with hepatocellular carcinoma opened new avenues, RNAi-based therapeutics for cancer therapy still remains a major challenge.^30^

In this study, we demonstrated that the apoptotic effect of miR-491-5p in ovarian cancer cells is related to both BCL-X_L_ inhibition and BIM stabilization through inhibition of EGFR signalling, as long as downstream effectors are not constitutively activated.

During the course of this study, it was reported that miR-491-5p is directly targeting BCL-X_L_ in various cell types.^22,23^ We show here, for the first time, that the EGFR is a direct target of miR-491-5p and that its downregulation by this miRNA subsequently leads to downstream AKT and MAPK signalling pathways inhibition. It should be noted that while the manuscript of this article was under review, Li *et al.*^31^ observed that miR-491-5p also targets EGFR in glioblastoma. Interestingly, other studies have also recently shown that miR-491-5p could inhibit AKT or/and MAPK pathways in different cellular context.^23,24,27^ For example, in breast cancer cell lines, miR-491-5p decreases AKT and ERK expression and activity and indirectly downregulates HER2.^24^ However, in this model, EGFR has not been studied, and the decreased expression of AKT and MAPK suggests that the involved mechanisms could be different. Otherwise, a recent study described a regulatory signalling pathway of OSCC metastasis consisting of miR-491-5p and its downstream target, GIT1, which functions as a scaffold for EGF-induced ERK activation.^27^ In both studies, it would be interesting to analyse whether the decrease in activity of these downstream signalling cascades could be related to EGFR inhibition by miR-491-5p.

We demonstrated that BIM induction is critical for induction of apoptosis by miR-491-5p in ovarian carcinoma. Several lines of evidence also show an important role of BIM in targeted therapies-induced apoptosis.^18^ For example, EGFR inhibition with erlotinib or gefitinib leads to an increased expression of BIM that is critical for induction of apoptosis in cancer cells expressing activated mutant EGFR.^32,33^ Both AKT and MAPK survival pathways are known to negatively regulate BIM stability.^18^ Consistent with these observations, EGFR inhibitors (cetuximab, gefitinib, erlotinib) or the pharmacological inhibition of both AKT and MAPK pathways induces BIM stabilization in IGROV1-R10 cells. However, although both conditions induced BIM expression at the same extent as miR-491-5p, they failed to induce apoptosis, suggesting that BIM induction is not sufficient to trigger cell death. It has been shown that BIM activity required its release from various sequestrators, including anti-apoptotic BCL2 family members, to be able to directly activate BAX and BAK.^17,18,34^ However, it could also be considered that its binding to anti-apoptotic proteins participate in the release of BAX/BAK from their anti-apoptotic sequestrators. In these conditions, BIM could exert a dual role in the maintenance of a global pool of activated BAX/BAK proteins able to homodimerize and induce cytochrome c release from the mitochondria. Because concomitant AKT and ERK inhibition did not modulate BCL-X_L_ expression, whereas miR-491-5p downregulated it, miR-491-5p-mediated apoptosis could be related to the release of BIM from BCL-X_L_. Indeed, the combination of inhibitors of AKT and MAPK signalling pathways or cetuximab with a siRNA targeting BCL-X_L_ led to apoptosis, mimicking what was observed with miR-491-5p. In good agreement with the literature,^35^ we observed that in cells expressing high levels of BCL-X_L_ and MCL1 (as IGROV1-R10), the fraction of BIM bound by MCL1 is very low at basal level. In contrast, when BCL-X_L_ is downregulated by miR-491-5p treatment, a huge fraction of BIM was sequestered by MCL1, suggesting a shuttling of BIM sequestration between BCL-X_L_ and MCL1, depending on their relative expression levels. Thus, in a state that is referred to as ‘primed' for death,^5^ where multidomains have been previously activated and sequestered by BCL-X_L_ and MCL1, both miR-491-5p-mediated downregulation of BCL-X_L_ and inhibition of MCL1 through BIM would lead to the release of multidomains, thereby allowing them to multimerize and trigger apoptosis as suggested by others.^6,36^ However, it could be added that, owing to a high level of BIM observed in response to miR-491-5p, a significant amount of BIM could remain in the cytosol and contribute to maintain a high level of activated BAX/BAK. Altogether, the molecular basis underlying apoptosis in these models seems to be related to the balance between pro-survival BCL-2-like and BH3-only proteins that exists in the cancer cells.

Otherwise, it was recently shown that BIM and PUMA could cooperate to induce cell death in several cancer cell lines in response to EGFR pathways inhibition.^37^ However, the absence of induction of PUMA by miR-491-5p as well as the almost complete protection from miR-491-5p-induced apoptosis by a siRNA targeting BIM suggested that PUMA does not play a major role in miRNA-mediated cell death, at least in IGROV1-R10 cells.

Several studies have highlighted the importance of EGFR pathway in ovarian carcinoma leading to the use of EGFR inhibitors in clinical trials. Unfortunately, EGFR-directed therapies as single agents have yielded disappointing results.^38^ Two situations could be distinguished. First, EGFR and its downstream signalling pathways are functional and EGFR inhibition leads to the accumulation of dephosphorylated BIM. This situation is observed in IGROV1-R10 cells, apoptosis requiring in this case the release of BIM from BCL-X_L_ obtained by the use of a BH3-mimetic molecule such as ABT-737. Second, downstream alterations leading to constitutive activation of AKT and MAPK signalling pathways impede EGFR inhibitor activity. In this case, apoptosis will require both the direct inactivation of these pathways by specific inhibitors leading to BIM accumulation and its desequestration from BCL-X_L_ by a BH3-mimetic molecule, as observed in SKOV3 treated with the combination BEZ235/CI-1040/ABT-737.^21^ In both situations, it should be noticed that BH3-mimetic molecules are helpful to sensitize ovarian carcinoma cells to the induction of BIM mediated by the inhibition of EGFR cell signalling pathways. Accordingly, recent preclinical studies indicate that combining Navitoclax (the orally bioavailable analogue of ABT-737) with targeted therapies can be very efficient for the treatment of various solid tumors.^6^

In conclusion, through this study, we proposed that the deciphering of miRNAs targets and associated cell signalling pathways may lead to the rational proposition of combination targeted therapies. Indeed, as it is well accepted that miRNAs control functional organized networks,^39^ it is tempting to speculate that the nodes or hubs of important regulatory pathways targeted by the miRNAs might constitute pertinent targets for therapeutic intervention. Our work highlights the utility of functional studies to decipher the anticancer effects of miRNAs, particularly when combined with an analysis of the involved signalling pathways. These approaches may facilitate the identification of effective drug combinations and new relevant therapeutic targets and finally enable the proposition of new clinical trials. In this context, this study highlights the interest of the combination of an EGFR inhibitor with a BH3-mimetic molecule in two ovarian cancer cell lines (IGROV1-R10 and OVCAR-3) and suggests that this strategy might prove useful in improving the therapeutic care of ovarian carcinoma.

## Materials and Methods

### Ovarian cancer cell lines

The cisplatin-resistant IGROV1-R10 cell line was obtained from IGROV1 cell line as previously described.^8^ The cisplatin-resistant SKOV3 cell line was obtained from ECACC (Sigma Aldrich, St Quentin-Fallavier, France). The cisplatin-resistant OVCAR-3 cell line was obtained from ATCC (LGS Standards, Molsheim, France). They were grown in RPMI-1640 supplemented with 2 mM Glutamax and 10% fetal calf serum, 20 mM HEPES and 33 mM sodium bicarbonate (Fisher Scientific Bioblock, Illkirch, France).

### Drugs

The PI3K inhibitor (LY294002), the MEK inhibitor (CI-1040), the dual PI3K/mTOR inhibitor (NVP-BEZ235), the BH3-mimetic molecule (ABT-737) and the EGFR TKIs (erlotinib and gefitinib) were purchased from Selleck Chemicals (Munich, Germany). Stock solutions were prepared in DMSO according to the manufacturer's instructions.

### miRNA target predictions

The following algorithms were used: Targetscan 5.1 http://www.targetscan.org, Diana http://www.diana.cslab.ece.ntua.gr and miRanda http://www.microrna.org

### Transfection

miRNA mimics and siRNA were purchased from Dharmacon (ThermoScientific, Illkirch, France) or Eurogentec (Liege, Belgium). miRNA (miRNA mimic negative control #1, CN-001000-01, noted miR-Ctrl) or siRNA (control siRNA duplex negative control, SR-CL000-005, noted siRNA-Ctrl) that have been shown to target any known human mRNA were used as negative controls. siRNA guide sequences targeting indicated genes are as follows: BCL-X_L_ (5′-cuacgcuuuccacgcacag-3′, noted siRNA-BCL-X_L_), MCL1 (5′-uguuuagccacaaaggcac-3′, noted siRNA-MCL1) and BIM (5′-uaacagucguaagauaacc-3′, noted siRNA-BIM). Exponentially growing cells were seeded at 250 000 cells per 25 cm^2^ flask. Twenty-four hours after seeding, mi/siRNA duplexes were diluted in OptiMEM (Life Technologies, Saint Aubin, France) and cells were transfected using INTERFERin (Polyplus-Transfection, Strasbourg, France) with indicated mi/siRNA to a final concentration of 20 nM.

### Real-time cell analysis (xCELLigence)

Real-time growth curves monitoring was performed with the Real-Time Cell Analyzer multi-plate instrument, using the xCELLigence System (ACEA, Ozyme, Saint Quentin en Yvelines, France). This system monitors cellular events in real-time by measuring electrical impedance across interdigitated micro-electrodes integrated into the bottom surfaces of 96-well E-plates VIEW (Ozyme). These electrodes measure CI based on impedance. CI correlates with the area of cells attached to the bottom of the plate.^40^ CI values are derived and displayed in the plot. Briefly, 7 × 10^3^ cells/well IGROV1-R10 or 3.5 × 10^3^ cells/well SKOV3 were plated in 96-well E-Plate View and placed onto the Real-Time Cell Analyzer multi-plate located inside a tissue culture incubator. Cells were left to grow for 24 h before treatment and impedance was continuously measured until the end of the treatment. Standard deviations of well replicates were analysed with the RTCA software.

### RNA isolation and TaqMan gene assays

Total RNA was isolated from transfected cells using Trizol (Invitrogen). RNA quantity and quality were assessed using the NanoDrop 2000 spectrophotometer (Thermo Scientific, Palaiseau, France). The expression of BCL-X_L_ and GAPDH transcripts were determined by RT-qPCR. The first strand cDNA was synthesized using Omniscript reverse transcriptase kit (Qiagen, Courtaboeuf, France) with random hexamers. Corresponding custom designed (forward, 5′-tgcgtggaaagcgtagacaa-3′ reverse, 5′-aggtaagtggccatccaagct-3′ probe 5′-FAMagatgcaggtattggtgMGB-3′) and inventoried (Hs99999905_m1 for GAPDH) TaqMan Gene Expression Assays were used (Applied Biosystems, Life Technologies). GAPDH was used as a housekeeping reference gene for normalization. For expression quantification, PCR amplification was performed in triplicate in an ABI 7500 Fast Real-time PCR system (Applied Biosystems, Life Technologies). Data are representative of three independent experiments performed in triplicate and no-template controls were included for each assay. The 2^−ΔΔCq^ method was used to calculate relative changes in gene expression determined from real-time quantitative PCR experiments.^41^ Data are presented as the percentage of untreated cells and as means±S.D.

### Western blotting

Cells were scraped and lysed in 60 *μ*l ice-cold RIPA lysis buffer. After 30 min on ice, lysates were clarified (13 000 × *g*, 4 °C, 10 min), and protein concentrations were determined using the Bradford assay (Bio-Rad, Marnes-la-Coquette, France). Equal amount of proteins (20 *μ*g) were separated by SDS-PAGE on a 4–15% gradient polyacrylamide gel (Bio-Rad) and transferred to PVDF membranes using the Trans-Blot Turbo Transfer system (Bio-Rad). Protein levels were then analysed by immunoblotting with antibodies from Cell Signaling Technology (CST, Ozyme) unless otherwise indicated according to the manufacturer's instructions. The CST antibodies were the following: total AKT (9272S), phospho-AKT Ser473 (4060), phospho-AKT Thr308 (9258S), BCL-x_L_ (2764S), BIM (2819S), total and cleaved caspase-3 (9662S), total and cleaved caspase-9 (9502S), EGFR (4267P), total ERK (9102S), phospho-p44/42 MAPK (Erk1/2) (Thr202/Tyr204) (4370), PARP (9542S), PUMA (12450S), and phospho-p70 S6 Kinase (Thr389) (9205S). MCL1 (S19) was from Santa Cruz Biotechnology (Le Perray en Yvelines, France). Appropriate horseradish-peroxidase-conjugated secondary antibodies (CST or GE HealthCare Europe GmbH, Velizy-Villacoublay, France) were used and signals were detected using enhanced chemiluminescence (GE HealthCare Europe GmbH). Blots were also hybridized with *β*-actin (Eurobio, Courtaboeuf, France) or *α*-tubulin (Sigma-Aldrich) monoclonal antibodies to control protein loading. Each immunoblot is representative of three distinct experiments. In some cases, protein expression was measured by quantifying the density of immunoblots bands adjusted to *β*-actin (image analysis software ImageJ).

### Phosphatase treatment

Exponentially growing cells were transfected or not by miR-491-5p or miR-Ctrl as described above. After 48 h incubation at 37 °C, the cells were detached from the plates by trypsinization, pelleted, washed with PBS and lysed on ice with a RIPA buffer without phosphatase inhibitors. Total protein from cell lysates was quantitated using Bradford Protein Assay. Then, cell lysates were diluted in cold calf intestine phosphatase buffer and treated with calf intestine phosphatase (Promega, Charbonnières, France) at a ratio of 1 U/*μ*g protein for 45 min at 37 °C. Reaction was stopped by adding Na_3_VO_4_ at 10 mM and proteins were analysed by western blotting.

### Immunoprecipitation

Total cell lysates were prepared in ice-cold lysis buffer (30 mM HEPES, 100 mM KCl, 20 mM NaCl, 2 mM MgCl_2_, 5% glycerol, 2% heptanetriol, 0.5% laurylmaltoside, 5 *μ*M GDP, 1 *μ*M microcystine, 1 mM sodium orthovanadate and a complete mixture of protease inhibitors (Roche, Meylan, France)). Proteins (500 *μ*g) were incubated with BIM monoclonal antibody (2933S, CST) precoated on protein G-Sepharose (GE HealthCare Europe GmbH) overnight at 4 °C under gentle agitation. After centrifugation and three washes in lysis buffer, the resulting protein complexes and the total cell extracts were separated by SDS-PAGE. The expression of MCL1 protein was then analysed by standard immunoblotting.

### Nuclear morphology

Detached and adherent cells were pooled and applied to a polylysine-coated glass slide by cyto-centrifugation, fixed with a solution of ethanol/chloroform/acetic acid (6 : 3 : 1) and then incubated with 1 *μ*g/ml DAPI solution (Roche), washed in water and mounted in Mowiol (Calbiochem, Merck4Biosciences, Nottingham, UK).

### Cell cycle analysis

Cells were detached by trypsinization, washed with PBS, fixed in 70% ethanol and stored at −20 °C until analysis. Fixed cells were centrifuged (2000 r.p.m., 5 min) and incubated for 30 min at 37 °C in PBS. After centrifugation, cells were resuspended and stained with propidium iodide using the DNA-Prep Coulter Reagent Kit (Beckman Coulter, Villepinte, France) and were analysed using an EPICS XL flow cytometer (Beckman Coulter). Computerized gating was applied on the side and forward scatter to exclude small debris and on a pulse width and integral peak of red fluorescence to eliminate aggregates. The data were analysed by Expo32 acquisition software (Beckman Coulter).

### Analysis of apoptosis by flow cytometry

Adherent and detached cells were pooled, washed and labeled with annexin-V-FITC and propidium iodide using the annexin-V-Fluos Staining Kit (Roche Applied Science, Meylan, France) according to manufacturer's instructions. Samples were acquired with a Gallios Flow Cytometer and analysed with Kaluza software (Beckman Coulter) to determine the percentage of cells displaying annexin V staining.

### Plasmid constructs

The Bcl-X_L_ 3′-UTR (NM_138578, +1069 to +2534) and EGFR 3′-UTR (NM_005228, +3875 to +5552) were amplified by PCR using restriction-enzyme-containing primers (Supplementary Figure S6). After amplification, specific enzymatic restriction and purification, the BCL-X_L_ and EGFR 3′-UTRs were inserted downstream the luciferase ORF by ligation into the linearized reporter plasmid pMIR-REPORT (Ambion, Life Technologies). These clones will be referred to as pMIR-XL/3′-UTRLuc and pMIR-EGFR/3′-UTRLuc. Mutant vectors containing 3′-UTR with miR-491-5p-deleted binding sites were generated by PCR using specific primers designed with the QuickChange primer design software (Supplementary Figure S9) and the QuickChange XL Site-Directed Mutagenesis Kit (Stratagene, Agilent Technologies, Massy, France) according to the manufacturer's instructions.

### Luciferase miRNA target reporter assay

Briefly, SKOV3 cells were co-transfected in six-well plate using JetPRIME (Polyplus Transfection) with 100 ng of a reporter plasmid containing wild-type 3′-UTR or deleted sites in the 3′-UTR and 10 ng of a *Renilla* luciferase plasmid as an internal control for transfection efficiency (pRL-TK vector, Promega) and with 20 nM of miR-491-5p or miR-Ctrl using INTERFERin. *Firefly* and *Renilla* luciferase activity was assayed 24 h after transfection with Dual-Luciferase Reporter Assay System (Promega) and measured with a luminometer Centro LB 960 (Berthold, Thoiry, France). Each assay contained three technical replicates and the assays were repeated at least three times.

### Statistical analysis

All experiments were carried out in triplicate parallel instances and independently repeated at least three times. Data were analysed with Microsoft Excel (Microsoft France, Issy-les-Moulineaux, France) and statistical significance was assessed by means of two-tailed unpaired Student's *t*-test (*P<0.05; **P<0.001).

## Abbreviations

EFGR: epidermal growth factor receptor
miRNA: microRNA
siRNA: small interfering RNA
3′-UTR: 3′-untranslated region
