# Supplementary material for: miR-491-5p-induced apoptosis in ovarian carcinoma depends on the direct inhibition of both BCL-XL and EGFR leading to BIM activation
Source: Cell Death Dis. 2014 Oct 9;5(10):e1445–. doi: 10.1038/cddis.2014.389 (PMC4649504; doi:10.1038/cddis.2014.389)
Supplement: Supplementary Figure S1 [file cddis2014389x1.pdf]

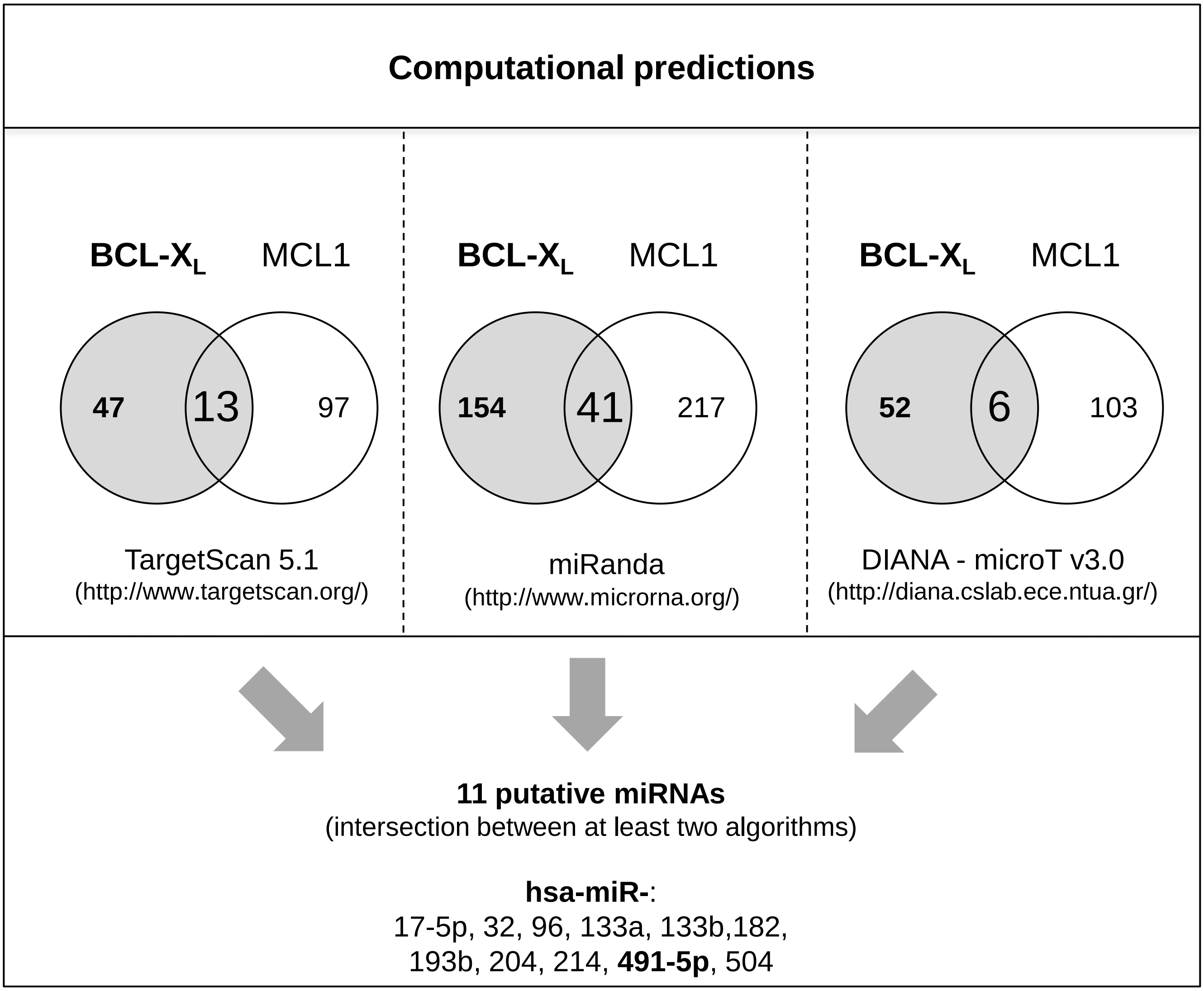

Supplementary Figure S1. Denoyelle et al.
