# Supplementary figures and images for: miR-491-5p-induced apoptosis in ovarian carcinoma depends on the direct inhibition of both BCL-XL and EGFR leading to BIM activation
Source: Cell Death Dis. 2014 Oct 9;5(10):e1445–. doi: 10.1038/cddis.2014.389 (PMC4649504; doi:10.1038/cddis.2014.389)

a

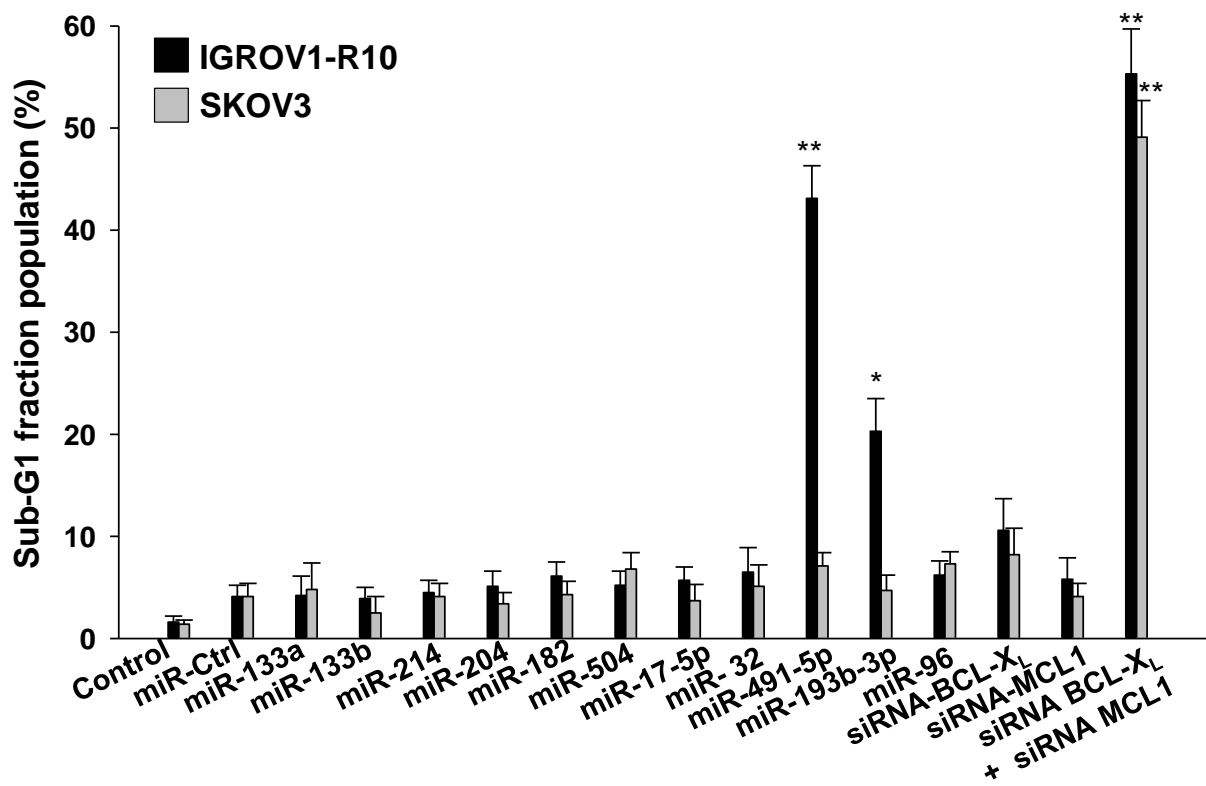

b

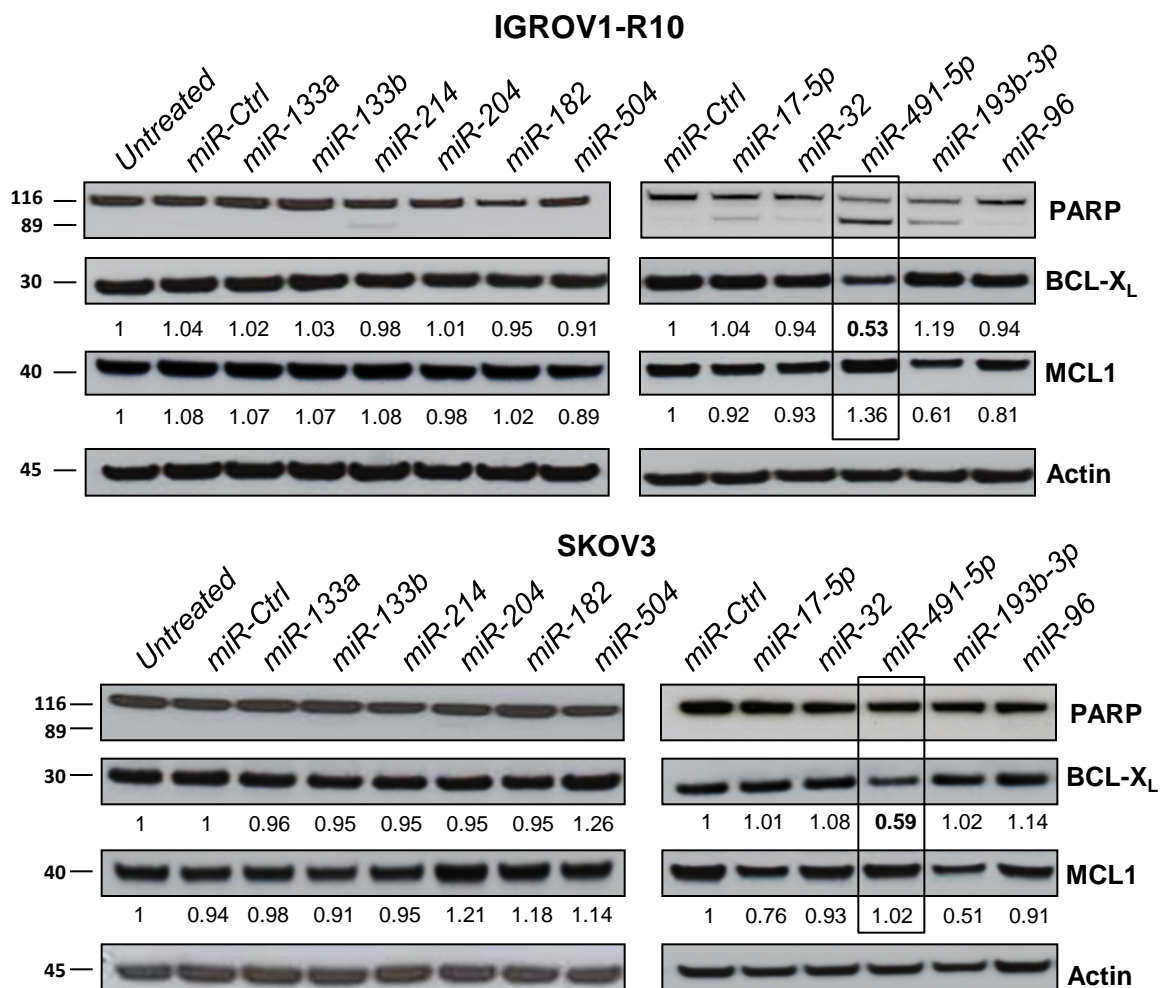

Supplement: Supplementary Figure S2 [file cddis2014389x2.pdf]

**a**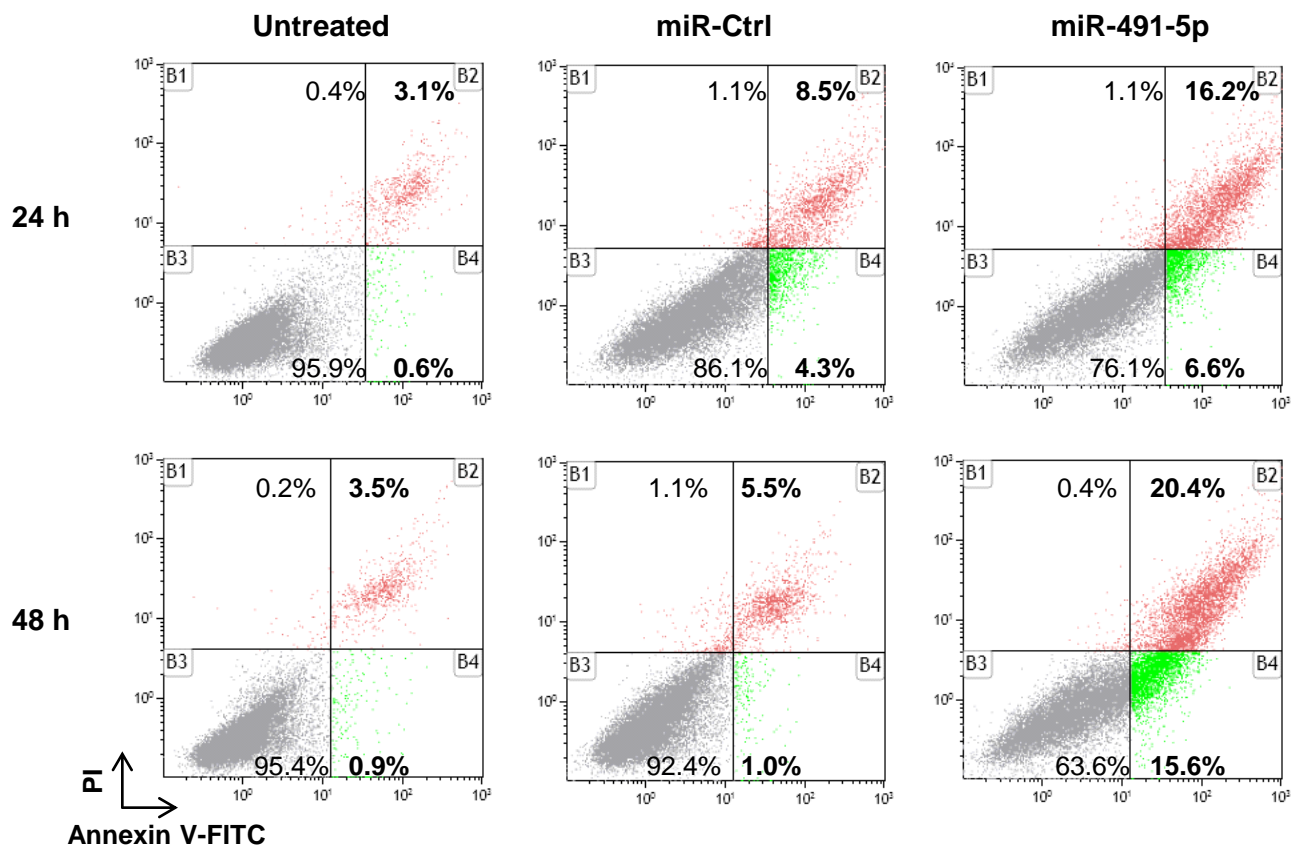**b**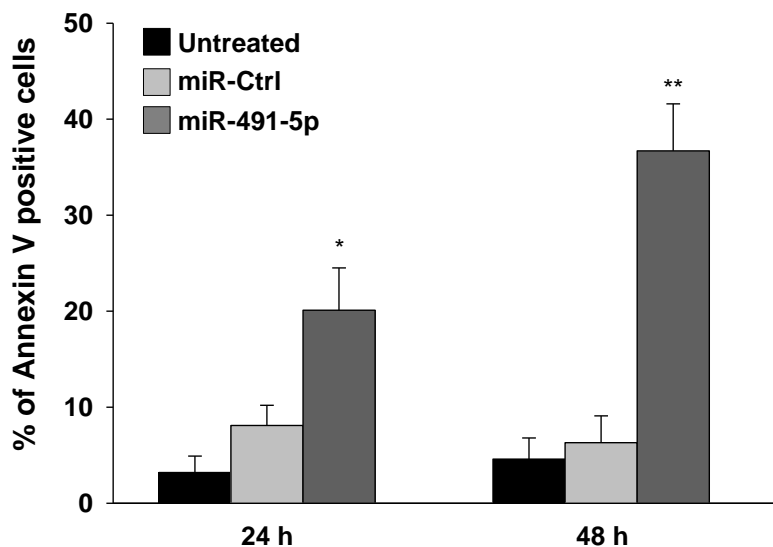

Supplement: Supplementary Figure S3 [file cddis2014389x3.pdf]

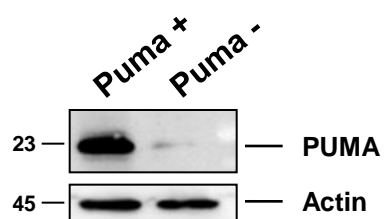

Supplement: Supplementary Figure S4 [file cddis2014389x4.pdf]

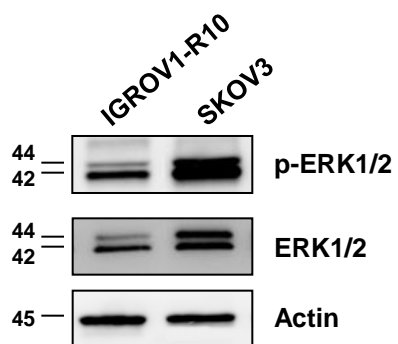

Supplement: Supplementary Figure S5 [file cddis2014389x5.pdf]

**a**

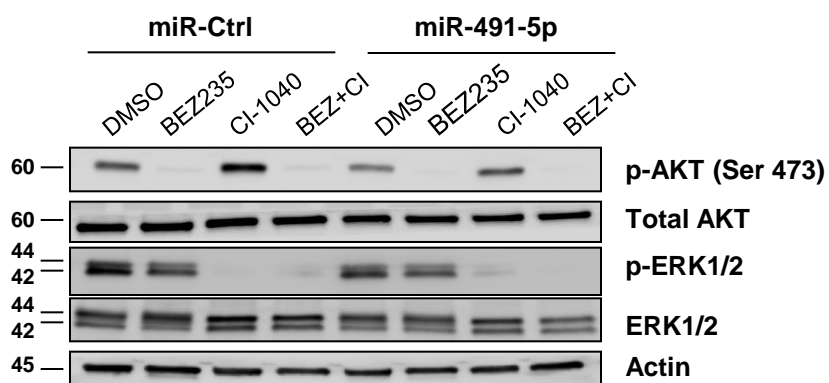

**b**

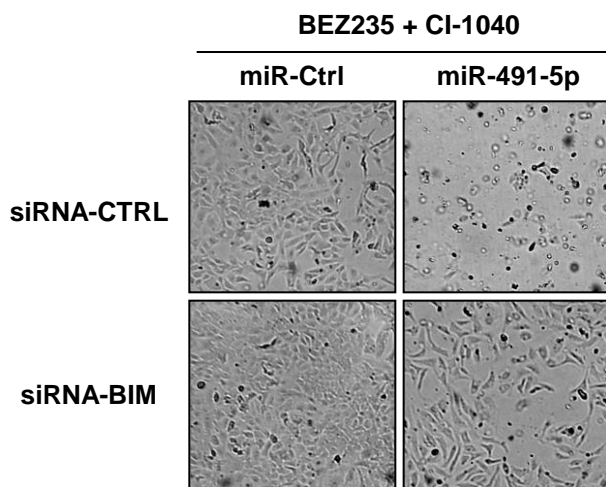

Supplement: Supplementary Figure S6 [file cddis2014389x6.pdf]

**a****IGROV1-R10**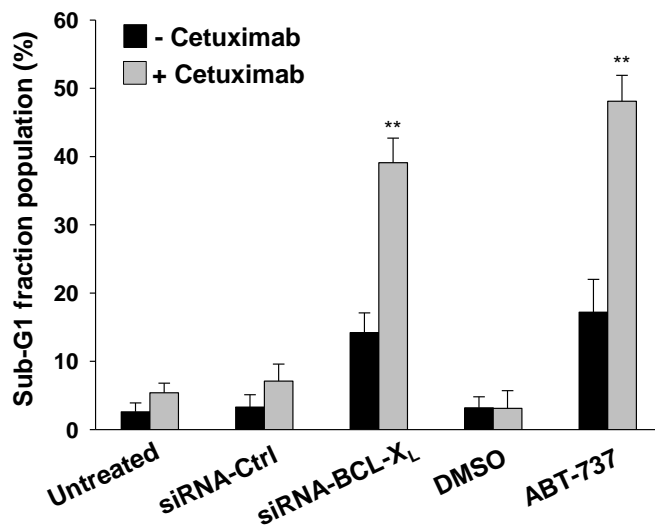**b**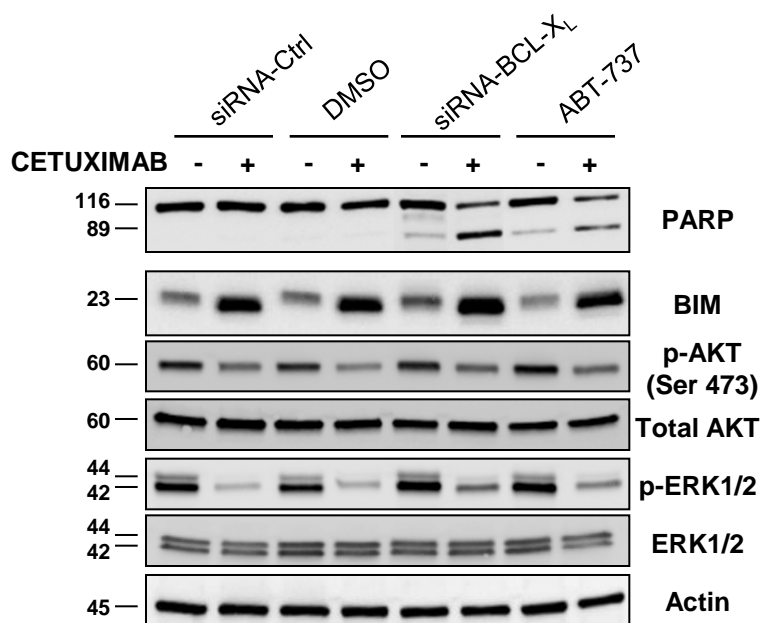**c**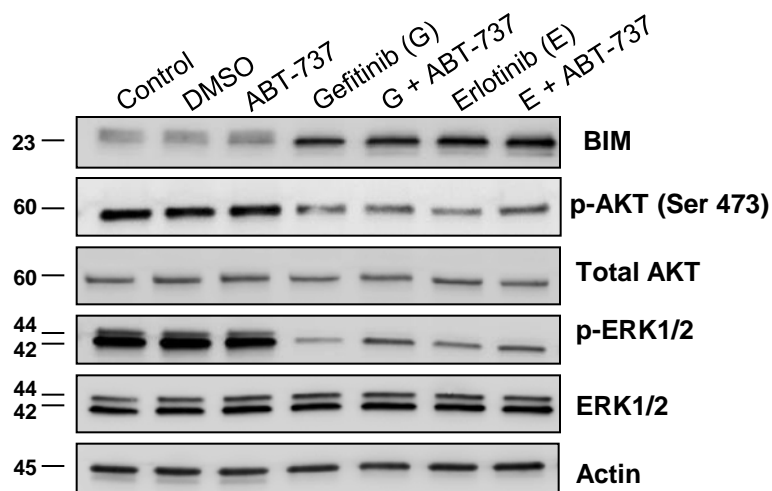

Supplement: Supplementary Figure S7 [file cddis2014389x7.pdf]

# SKOV3

**a**

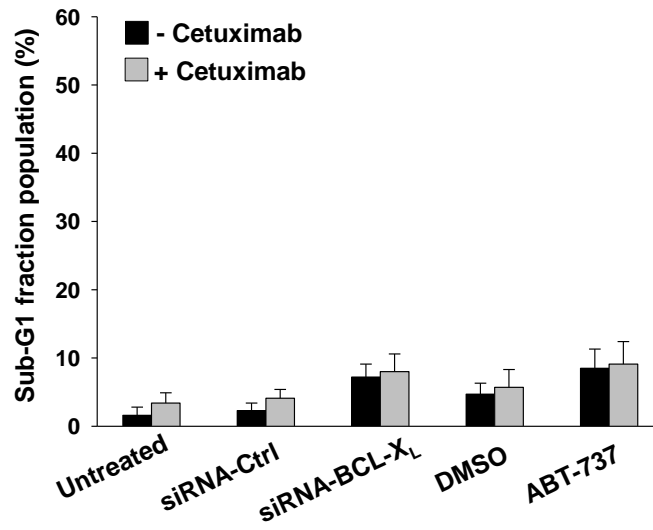

**b**

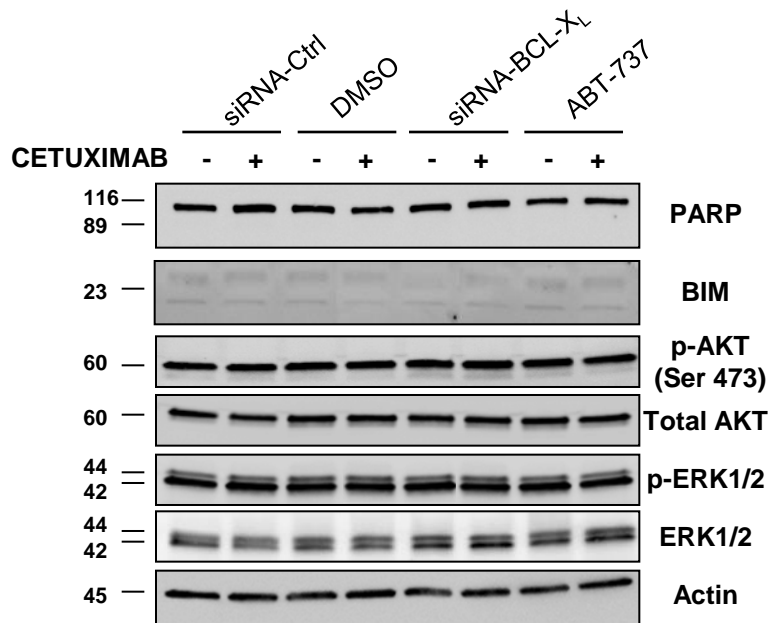

**c**

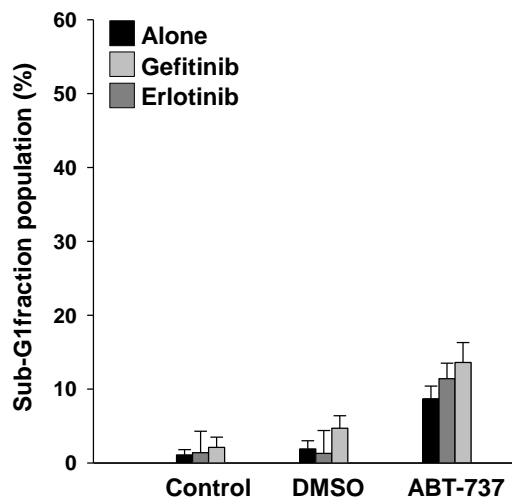

Supplement: Supplementary Figure S8 [file cddis2014389x8.pdf]
