# Supplementary material for: miR-491-5p-induced apoptosis in ovarian carcinoma depends on the direct inhibition of both BCL-XL and EGFR leading to BIM activation
Source: Cell Death Dis. 2014 Oct 9;5(10):e1445–. doi: 10.1038/cddis.2014.389 (PMC4649504; doi:10.1038/cddis.2014.389)
Supplement: Supplementary Figure S9 [file cddis2014389x9.pdf]

3’UTR		Primers for amplification	RE binding sites (underlined)
**BCL-X_L**	for:	5’-cgacgcgtccagacactgaccatc-3’	*Mlu*I
	rev:	5’-cccaagcttctgaactgcactttcac-3’	*Hind*III
**EGFR**	for:	5’-ccactagtgcatgaccacggaggatagt-3’	*Spe*I
	rev :	5’-cacgcgtattttcattgagacaaaaatcaaat-3’	*Mlu*I

Mutant name	Primers for miR-491-5p site deletion	matrixes
pMIR-XLDel1/3’UTRLuc	for: 5'-gctagttttctagaatttatcctcagttcccttggcctc-3'<br>rev: 5'-gaggccaagggaactgaggataaattctagaaaactagc-3'	pMIR-XL/3’UTRLuc
pMIR-XLDel2/3’UTRLuc	for: 5'-agggaacccaggttagaatgatcaattctgaggcc-3'<br>rev: 5'-ggcctcagaattgatcattctaacctgggttccct-3'	pMIR-XL/3’UTRLuc
pMIR-XLDel1.2/3’UTRLuc	for: 5'-agggaacccaggttagaatgatcaattctgaggcc-3'<br>rev: 5'-ggcctcagaattgatcattctaacctgggttccct-3'	pMIR-XLDel1/3’UTRLuc
pMIR-EGFRDel/3’UTR	for: 5’-gaagaaacggaggggatggaattctagactgacttgtt-3’<br>rev: 5’-aacaagtcagtctagaattccatcccctccgtttcttc-3’	pMIR-EGFR/3’UTRLuc

Supplementary Figure S9. Denoyelle et al.
